# Heart failure: a prevalence-based and model-based cost analysis

**DOI:** 10.3389/fcvm.2023.1239719

**Published:** 2023-12-01

**Authors:** Zahra Mahmoudi, Maryam Chenaghlou, Hossein Zare, Naser Safaei, Mahmood Yousefi

**Affiliations:** ^1^ Department of Health Economics, School of Management and Medical Informatics, Tabriz University of Medical Sciences, Tabriz, Iran; ^2^ Cardiovascular Research Center, Tabriz University of Medical Sciences, Tabriz, Iran; ^3^ Department of Health Policy and Management, Johns Hopkins Bloomberg School of Public Health, Johns Hopkins University, Baltimore, MD, United States

**Keywords:** cost-of-illness, economic burden, heart failure, Markov models, financial burden

## Abstract

**Introduction:**

Heart failure (HF) imposes a heavy economic burden on patients, their families, and society as a whole. Therefore, it is crucial to quantify the impact and dimensions of the disease in order to prioritize and allocate resources effectively.

**Methods:**

This study utilized a prevalence-based, bottom-up, and incidence-based Markov model to assess the cost of illness. A total of 502 HF patients (classes I–IV) were recruited from Madani Hospital in Tabriz between May and October 2022. Patients were followed up every two months for a minimum of two and a maximum of six months using a person-month measurement approach. The perspective of the study was societal, and both direct and indirect costs were estimated. Indirect costs were calculated using the Human Capital (HC) method. A two-part regression model, consisting of the Generalized Linear Model (GLM) and Probit model, was used to analyze the relationship between HF costs and clinical and demographic variables.

**Results:**

The total cost per patient in one year was 261,409,854.9 Tomans (21,967.21 PPP). Of this amount, 207,147,805.8 Tomans (17,407.38 PPP) (79%) were indirect costs, while 54,262,049.09 Tomans (4,559.84 PPP) (21%) were direct costs. The mean lifetime cost was 2,173,961,178 Tomans. Premature death accounted for the highest share of lifetime costs (48%), while class III HF had the lowest share (2%). Gender, having basic insurance, and disease class significantly influenced the costs of HF, while comorbidity and age did not have a significant impact. The predicted amount closely matched the observed amount, indicating good predictive power.

**Conclusion:**

This study revealed that HF places a significant economic burden on patients in terms of both direct and indirect costs. The substantial contribution of indirect costs, which reflect the impact of the disease on other sectors of the economy, highlights the importance of unpaid work. Given the significant variation in HF costs among assessed variables, social and financial support systems should consider these variations to provide efficient and fair support to HF patients.

## Introduction

Heart failure (HF) is a complex syndrome where the heart fails to properly pump blood, leading to a lack of oxygen and nutrients for the tissues. It is characterized by symptoms such as shortness of breath, fatigue, chest pain, and fluid retention (1, 2). HF is a significant global public health issue (3, 4), affecting over 64 million people worldwide (5). Its prevalence is increasing in developing countries, including Iran (6, 7), where it accounts for 25% of cardiac ward hospitalizations (8, 9). The re-hospitalization rate for HF patients is around 40% (10). This increase in prevalence (11) is due to factors such as an aging population (12), improved survival rates for conditions like myocardial infarction and diabetes, lifestyle changes, and advancements in diagnostic technologies (13–16). It imposes heavy costs and a significant economic burden on patients, their families, and society (17–19). It accounts for a large part of the growing costs of the health system. Hospitalization is the most significant part of it (14, 20–22). The lack of resources and increasing social expectations have made the prioritization of available resources an integral part of health care delivery systems.

Gaining knowledge and information about the level and dimensions of the impacts of diseases is necessary for planning and prioritizing resource allocation for disease control (23–25). Understanding the impacts and dimensions of diseases and expressing them monetarily is crucial for efficient resource allocation (26). Quantifying the economic burden of HF can aid decision-makers in allocating resources, reflecting the financial burden on society, and estimating the benefits of preventive measures (27). Various studies have employed different methods to explore the financial burden of diseases in healthcare systems, depending on the purpose of cost analysis (28–30). The cost of illness (COI) method is commonly used to evaluate the impact of diseases on populations, considering both direct and indirect costs (25, 26, 31). The ultimate goal of a COI study is to present objective evidence needed for policymaking, designing, and managing health programs. By comparing the costs of HF in different countries with varying health systems, we can gain insights into the economic impacts of different health policies and programs (25). This study aims to analyze the economic costs of heart failure.

## Methods

The present study utilized a prevalence-based and incidence-based cost-of-illness approach. The prevalence-based analysis was conducted using a bottom-up approach, while the incidence-based analysis employed Markov modeling. Data for this study were collected from May to October 2022. A total of 502 heart failure patients with diagnostic codes (I50.1, I50.2, I50.3, I50.4, and I50.8) according to the ICD-10 and classified as classes I, II, III, or IV based on the New York Heart Association (NYHA) classification system were selected using a systematic sampling method. These patients were selected among those referred to Shahid Madani Hospital of Tabriz, with ethics approval IR.TBZMED.REC1401.361. The sample consisted of patients who had visited the hospital as inpatients or outpatients within the past two years. Shahid Madani Hospital is a specialized public center for heart patients in the northwest of Iran. Patients were followed up every two months for a minimum of two months and a maximum of six months using a person-month measure. This approach was applied to all types of included costs, except for inpatient costs. For inpatient costs, we asked whether patients had been admitted to the hospital in the last year due to their HF disease and this hospitalization rate was applied for one-year period. Additionally, we calculated the average cost of admission per HF patient using hospital bills.

The final outcome of the cost analysis was calculated as an annual cost per patient. To achieve this, the monthly cost of each patient (using person-month average) was multiplied by 12 to obtain the annual cost. This study estimates the costs of heart failure from a societal perspective, considering both direct and indirect costs. Three stages of cost identification, measurement, and valuation were implemented to calculate these costs. The value of resources used was determined based on private sector tariffs, and indirect costs were calculated using the human capital approach, which measures productivity lost due to absenteeism, premature death, presenteeism (low productivity), unpaid work, and job change.

Data for direct costs were collected using the Medical Consumption Questionnaire and data for indirect costs were collected using the Productivity Costs Questionnaire (32), both of which are registered questionnaires from Erasmus University in the Netherlands. Direct cost data were extracted from the Shahid Madani Hospital database and through telephone interviews. Mean hospitalization costs were obtained from the hospital database, while costs related to medication and outpatient services were obtained through telephone interviews. Only costs specifically related to heart failure were considered in the estimation of direct costs. Costs related to underlying diseases were not included. Indirect costs were also obtained through telephone interviews.

### Estimating incidence-based costs using modeling

**Figure 1:**
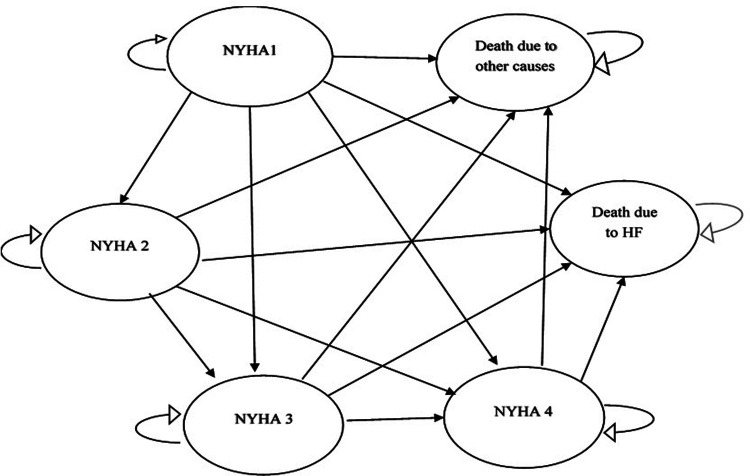
The Markov chain used in the model. NYHA = New York Heart Association Heart Failure Class.

In addition to estimating annual costs, this study also estimated the costs of heart failure over a patient’s lifespan using a modeling approach. A Markov model with six states was developed for this purpose. The states of illness were defined based on the NYHA classification method and included NYHA1, NYHA2, NYHA3, NYHA4, as well as two death states (death due to other causes and death due to heart failure). The time horizon of the model was set at 25 years with one-year cycles. Transition probabilities between different states were estimated based on the effectiveness of common ACE treatments using relevant studies (33). Mean costs for each state in each year were derived from the data collected in the prevalence-based analysis. Finally, the mean cost per patient over their lifetime was calculated after implementing the model. (See Figure 1. The Markov chain used in the model. NYHA = New York Heart Association heart failure class.).

### The effect of clinical and demographic variables on the cost of heart failure

The study aims to analyze the economic costs of heart failure by considering the impact of clinical and demographic variables. To address the challenges of asymmetry of cost data, zero-cost, and censored data, a two-part regression model was used. This model includes two parts: the Probit model for discrete variable regression estimation and the Generalized Linear Model (GLM) for continuous variable regression estimation. The variables examined in the regression model include age, insurance status (having or not having basic insurance), class of disease, comorbidity, and gender. The goodness of fit of the model was assessed using the AIC index, and the regression coefficients were examined at a 95% level of significance. The regression analyses were performed in Stata_16 Software.

## Results

### Demographic and clinical characteristics of patients

Table 1 presents the demographic and clinical characteristics of the 502 heart failure patients included in the study. Out of the patients, 61.1% were male and 38.9% were female. The majority (91.2%) had basic insurance coverage, while 8.8% did not have any insurance. Among the patients, 61% had at least one underlying disease, such as diabetes, hypertension, hyperglycemia, kidney failure, lung disease, or thyroid issues. The remaining 39% did not have any underlying diseases. Additionally, 42.4% of the patients had supplemental insurance coverage, while 57.6% did not. The majority (72%) of the heart failure patients were heads of households. The mean age of the patients was 63.46 years, with a standard deviation of 14.27. The youngest patient was 8 years old, and the oldest patient was 95 years old.

**Table 1:** Demographic and clinical characteristics of patients.

Variable	Status	Frequency	Percent
HF	Class I	106	21.1
	Class II	189	37.7
	Class III	88	17.5
	Class IV	119	23.7
Gender	Male	307	61.1
	Female	195	38.9
Comorbidity	Yes	306	61
	No	196	39
Basic insurance	Yes	458	91.2
	No	44	8.8
Complementary insurance	Yes	213	42.4
	No	289	57.6
Marital status	Single	15	3
	Married	382	76.1
	Divorced	5	0.9
	Spouse died	100	20
Level of education	Illiterate	197	39.2
	Under & diploma	261	52
	Associate's degree—Bachelor's degree	30	6
	Master's degree—PhD	14	2.8
	Seminary education	0	0
State of residence	Local	261	52
	Non-local	241	48
Household Head	Yes	361	72
	No	141	28

### Estimating the annual costs of heart failure

Table 2 presents the estimated annual costs of heart failure per patient. The total annual cost per patient was calculated to be 261,409,854.9 Tomans. Direct costs accounted for 54,262,049.09 Tomans per patient, which included direct medical costs of 52,260,051.17 Tomans and direct non-medical costs of 2,001,997.923 Tomans. Indirect costs made up a larger share of the total costs at 207,147,805.8 Tomans per patient. Among the different classes, class IV had the highest cost at 332,555,849 Tomans per patient per year, while class II had the lowest cost at 184,586,207.1 Tomans per patient per year. Approximately 60% of the patients had been hospitalized in the last year, and hospitalization costs accounted for 20% of the total direct medical costs. Additionally, around 15% of the sample had visited the emergency department.

**Table 2:** Average and standard deviation of the annual costs of heart failure.

Variable			Mean (Toman)	Standard deviation (SD)
Average total annual cost			261,409,854.9	369,357,786.7
Average annual direct cost	Medical	Medicine	5,053,339.627	31,551,902.47
		Treatment	44,979,659.96	100,769,273.6
		Diagnostic	2,184,442.024	6,206,215.425
	Non-medical		2,001,997.923	5,283,430.999
Average annual indirect cost	Premature death		29,403,824.7	158,145,195.4
	Absenteeism		9,390,793.338	28,323,561.66
	Presenteeism		316,600.2596	2,292,269.639
	Job change		0	0
	Unpaid work		168,036,587.5	315,918,806
Average annual cost	Class I		284,221,375.3	484,971,996.5
	Class II		184,586,207.1	309,046,776.5
	Class III		302،719,797.7	345,431,304.9
	Class IV		332,555,849	331,530,122.6

**Figure 2:**
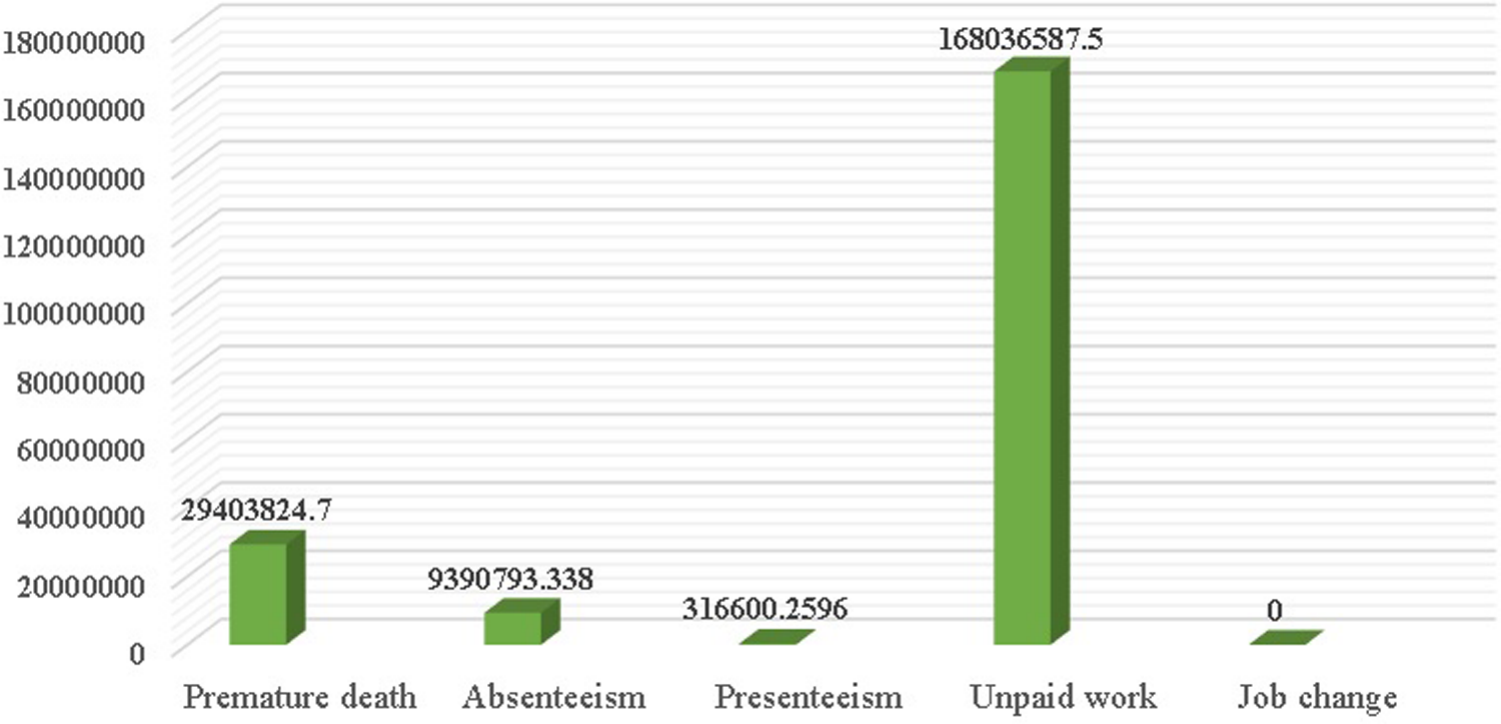
Indirect costs in heart failure patients.

Figure 2 illustrates the breakdown of indirect costs for heart failure patients. A significant portion of the costs incurred by patients is not merely due to medical treatment or medical costs but also due to the impact of the disease on their daily lives. The largest share of indirect costs was attributed to unpaid work, including household chores, child care, or voluntary social activities, which amounted to 168,036,587.5 Tomans per patient per year. The cost of premature death was estimated at 29,403,824.7 Tomans, while absenteeism cost 9,390,793.3380 Tomans. Presenteeism (low productivity) resulted in a cost of 316,600.2596 Tomans, and there were no costs associated with job changes.

**Figure 3:**
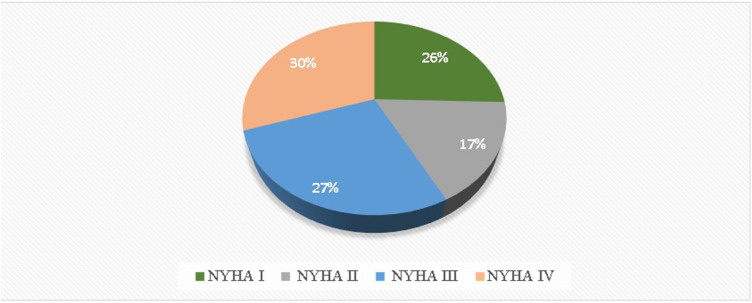
Annual average cost in disease states (class I, II, III, IV).

Figure 3 displays the average annual costs per patient in different disease states (class I, class II, class III, and class IV). Class IV had the highest annual cost per patient, accounting for 30% of the total cost, while Class II had the lowest cost at 17% of the total. Class I accounted for 26% of the total costs, and Class III accounted for 27%.

**Figure 4:**
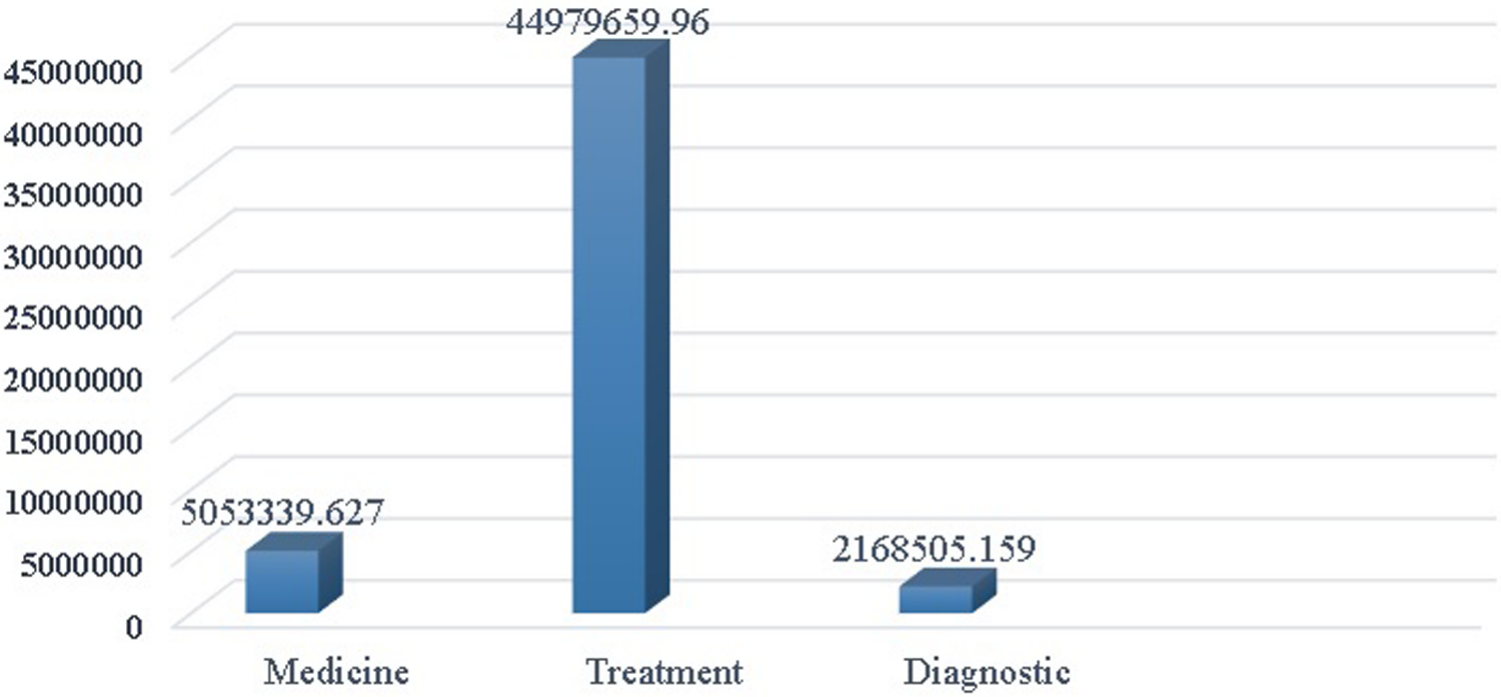
Annual direct medical costs in HF heart failure patients.

Figure 4 presents the distribution of direct medical costs. The majority (86%) of the total direct medical costs were attributed to treatment costs, including home care, visits, ambulance transportation, emergency department visits, and hospitalization. These costs amounted to 44,979,659.96 Tomans per heart failure patient per year. The lowest share (4%) of the total direct medical costs was associated with diagnostic costs, including imaging and laboratory services, which amounted to 2,184,442.024 Tomans per patient per year. The cost of medications was estimated at 5,053,339.627 Tomans per patient per year, accounting for 10% of the total direct medical costs.

**Figure 5:**
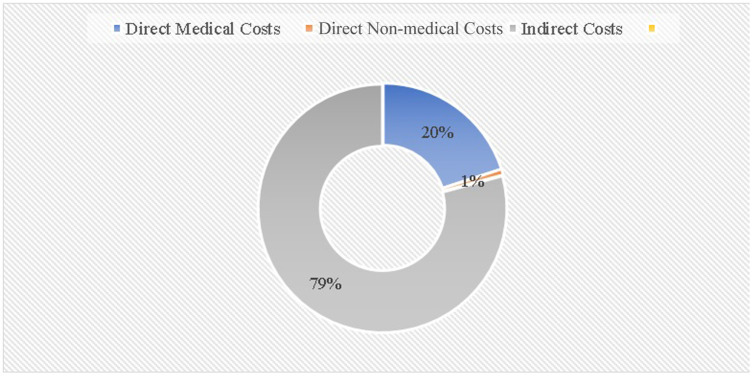
Annual direct costs (medical and non-medical) and indirect costs in heart failure patients.

Figure 5 illustrates the share of direct and indirect costs incurred by heart failure patients annually. The majority of these costs are attributed to indirect expenses, accounting for 79% of the total, followed by direct medical costs at 20%, and non-medical direct costs at approximately 1%.

### The lifetime cost of HF

The study utilized the incidence-based method to calculate the average costs for heart failure patients. This approach involved the use of a simulated cohort through the Markov model. The mean estimated cost for a heart failure patient was found to be 2,173,961,178 Tomans. The costs associated with each Markov model state, such as NYHA classes and premature death, were also determined. Specifically, the cost for class I was estimated at 471,146,883 Tomans, class II at 88,460,400 Tomans, class III at 51,001,097 Tomans, class IV at 517,348,089 Tomans, and premature death due to heart failure at 1,046,004,708 Tomans.

### Regression analysis

Table 3 presents the findings of a two-part regression analysis. Part A focuses on discrete regression and reveals that having insurance has a positive and significant impact on disease costs. Additionally, the age of the patient negatively and significantly affects the probability of zero costs at a 10% level. Gender, disease class, and comorbidity also exhibit a negative relationship with zero costs probability but do not reach statistical significance. In Part B (continuous), the GLM model was estimated. The analysis shows that costs decrease with increasing age; however, this relationship is not statistically significant. Gender is found to be a significant variable affecting costs, with females showing a negative relationship compared to males. Disease class has a positive and significant relationship with costs at a 5% level, indicating that costs significantly increase with higher disease class. Having a comorbidity increases costs, but this relationship is not statistically significant. Basic insurance does not significantly impact costs in GLM regression. The AIC and BIC indexes indicate the model’s good performance, and the model demonstrates relatively good predictive power, with the observed dependent variable (261 million Tomans) closely aligning with the predicted dependent variable (261 million Tomans).

**Table 3:** Two-part regression results (A. Discrete regression Probit, B. Continuous regression GLM).

A. Discrete Total Cost (Probit)	Coef	Std.Err	*Z*	*P* > IzI	Conf. interval	
Age	−.0108625	.0062124	−1.75	0.080	−.0230387	.0013136
Gender	−.1660696	.1780375	−0.93	0.351	−.5150166	.182877
Base insurance	.6149499	.2497434	2.46	0.014	.1254619	1.104438
Comorbidity	−.0449416	.1918412	−0.23	0.815	−.4209435	.3310603
Disease class	−.0960613	.0830214	−1.16	0.247	−.2587803	.0666576
Cons	1.988594	.4829774	4.12	0.000	1.041975	2.935212
B. Continuous regression (GLM)	Coef	Std.Err	*Z*	*P* > IzI	Conf. interval	
Age	−.0011812	.0046353	−0.25	0.799	−.0102663	.0079038
Gender	−.363384	.1385386	−2.62	0.009	−.6349148	.0918533
Base insurance	−.3233732	.241476	−1.34	0.181	−.7966576	.1499112
Comorbidity	.0577854	.1357614	0.43	0.670	−.2083021	.3238729
Disease Class	.1208097	.0578797	2.09	0.037	.0073676	.2342518
Cons	19.60896	.3910967	50.14	0.000	18.84242	20.37549

Log linkelihood = −9538.861943, AIC = 40.87735, BIC = −1835.199.

## Discussion

Heart failure is a significant health problem that imposes both health and financial burdens on patients and society. The results of this study provide a comprehensive analysis of the burden of heart failure across various dimensions. The study adopts a prevalence-based approach from a social perspective to estimate the average annual cost of heart failure. Unlike similar studies, this research takes into account both direct and indirect costs, including unpaid work costs. Moreover, in addition to the prevalence-based approach, the study also employs an incidence-based modeling method to estimate the costs of heart failure over the patient’s lifetime. This approach is less commonly used in studies focusing on this population. Furthermore, this study investigates the relationship between influential variables and their impact on the occurrence and changes in costs. An appropriate regression model is utilized to analyze these variables, and the subsequent findings for each topic are discussed in detail.

### Prevalence-based costing

The prevalence-based results reveal that the total cost per heart failure patient in one year is 261,409,854.9 Tomans. Indirect costs account for the majority of these costs, with unpaid work costs being the largest component. Direct costs, including direct medical and non-medical costs, make up a smaller portion of the total costs. The direct medical costs mainly consist of pharmaceutical, diagnostic, and treatment expenses. No study has been conducted on the economic burden of heart failure in the context of Iran using a similar design. However, several studies have been carried out on the overall burden of cardiovascular diseases. These studies did not specifically estimate the burden of heart failure and are not comparable to the current study in terms of methodological design, cost considerations, and reported outcomes. In one of those studies (6) authors estimated the cost of cardiovascular diseases, without considering the costs of unpaid works. As part of their sample, they had included some patients with heart failure, and the results showed that indirect costs accounted for 64% of the total costs. The study also estimated that hospitalization costs accounted for 35% of the costs. Since the study has not included the costs associated with unpaid work then the figures seem to be consistent with our study. A study by John Hong et al. (2017) investigated the medical costs related to heart failure in China. In that study, the annual medical costs per heart failure patient were 28,974 Yuan [7,212 PPP (2020)]. After updating the mentioned cost based on the inflation rate of China and then converting it to the international currency to compare the cost rates, the cost obtained in the Chinese study was slightly higher than the present study. This difference can be justified by the different conditions of the treatment protocols, structural differences in the service delivery system and health service tariffing method, and the conditions of access to services. The above-mentioned study did not investigate indirect costs, such as presenteeism due to heart failure disease (34).

Henkel Li et al. in South Korea estimated the prevalence and economic burden of heart failure disease. The mean cost per patient in 2014 was estimated at 1,414.0 dollars (1,896 PPP, 2020). It seems difficult to compare the total costs owing to the heterogeneity of the influential parameters. Medical costs have the largest share of the national burden (54.8 + 13.7% = 68.5%), followed by caregiver costs (13.2%), presenteeism costs due to premature death (10.8%), complications (4.2%), and transportation costs (3.4%), respectively (35). Since this study did not estimate the costs of presenteeism in the workplace and the cost of unpaid work, the ratio of direct costs was high compared to indirect costs. They also showed a pattern similar to our study in other dimensions, such as the share of direct non-medical costs and premature death costs.

Blazej estimated the indirect costs and financial outcomes of heart failure in Poland from 2012 to 2015. The results revealed that premature death accounted for 59.3%–63.4% of the total costs, presenteeism due to disability accounted for 11.1%–14.2% of the total costs, patient absenteeism accounted for 3.3%–4.0% of the total costs, and caregivers’ absenteeism accounted for 0.02% of the total costs (36). Since this study did not consider the cost of unpaid work, it is not logical to compare the cost components one by one. However, the distribution pattern of costs such as presenteeism and absenteeism of patients were similar to the present study.

Most of the articles investigated the economic burden of heart failure disease only in terms of limited costs such as direct or indirect costs. However, the present study considered all aspects of annual indirect costs per patient. One of the comprehensive characteristics of this study is considering the costs caused by unpaid work, which includes 64% of the total annual costs. The reason for high cost of this item is that heart failure affects the performance and ability of patients and increases their dependence on others. Also, since the majority of the heart failure patients were housewives or retired people, whose role in producing market value was low, unpaid activities accounted for a significant proportion of the total indirect cost. Generally, heart failure significantly affects the quality of life, performance, and productivity of people, which leads to the disability of patients and thus increases indirect costs.

### Incidence-based costs

The lifetime costs of heart failure patients were calculated using the incidence-based method in this study, along with the annual cost of the disease. Unlike some other studies that use real data and patient follow-up, this study estimated costs through modeling (simulation) using a Markov model with 6 health states. The average estimated lifetime cost for a patient was 2,173,961,178 Tomans. The model also considered lifetime costs associated with each Markov state, including NYHA classes and premature death due to heart failure. The highest proportion of costs was attributed to premature death due to heart failure, accounting for 48% of total costs, followed by class IV with 24%, class I with 22%, class II with 4%, and class III with 2%. While there are no studies specifically analyzing the costs of heart failure using modeling, some studies have examined the cost-effectiveness of interventions using a Markov model. The costs investigated in these studies can be considered consistent with the findings of this study.

In a study conducted by Yue Wu et al. in China, sacubitril/valsartan was compared to enalapril in heart failure patients. Using a Markov model, the study estimated the costs of the disease over a 10-year period. The mean cost of routine treatment (enalapril) was estimated at 4,014 US dollars over 10 years (37). However, this study only estimated direct costs and had a different perspective and time horizon, making it difficult to compare its results with those of the present study.

Similarly, Gaziano et al. used a two-state Markov model to estimate the medical costs of heart failure patients over 30 years. The total cost for this period was estimated at 83,303 US dollars (38). Again, due to differences in perspective and time horizon, it is not possible to directly compare these results with those of the present study.

Another study by Lorenzo Pradelli et al. examined the cost-effectiveness of using valsartan compared to the standard treatment method in Italy over a 10-year period. The cost of standard treatment was 87,899 euros (39). However, the heterogeneity of model parameters makes it difficult to compare these results with those of the present study.

### The results of the regression model

The present study utilized a two-part regression model. The Probit Model was used to investigate the probability of cost occurrence, in relation to examined clinical and socio-demographic variables, while the Generalized Linear Model (GLM) was used to evaluate the relationship of those variables with the level of cost variations. The results showed that gender, having basic insurance, and disease class significantly influenced the cost of the disease. On the other hand, comorbidity and age did not have a significant impact on costs. The model also successfully predicted the amount of cost, with the predicted amount being almost equal to 100% of the observed amount, indicating good predictive power.

Several studies have also examined the effects of these variables on the cost of heart failure. For example, a study by Teerapat et al. in several Asian countries found that age did not significantly affect costs in Malaysia and Thailand, which aligns with the findings of the present study. The lack of significant impact may be attributed to the limited variance in age range, as most participants were older individuals. Additionally, these studies reported that gender did not significantly affect costs. However, the present study revealed that gender does influence cost occurrence. The significant impact of gender on costs can be attributed to the non-inclusion of the monetary value of women’s housework, which has a significant influence on indirect costs. In Thailand, the role of disease class on costs showed a significant difference between class IV, class I, and class II, but no significant difference between class III and class IV. The results of our study were consistent with those of a study in Thailand in terms of the variation in the costs of disease classes. However, a study in Malaysia did not find a statistically significant difference in costs between disease classes, which may be due to the use of similar treatment protocols in the variables. Comorbidity did not have a statistically significant effect on costs in the studies conducted in Malaysia and Thailand, which is consistent with the findings of our study (40).

As expected, the presence or absence of basic insurance significantly affected the costs of the disease, with uninsured individuals experiencing lower costs (utilization). While there is limited information on the role of insurance in the costs of heart failure specifically, other studies have investigated its impact on costs in other diseases and reported statistically significant effects (41, 42).

## Conclusion

Heart failure is a common disease that has significant impacts on patients, including functional, physical, and psychological problems. This study found that heart failure imposes a substantial economic burden on patients, both in terms of direct costs and indirect costs. The indirect costs, which reflect the effects of the disease on other economic sectors, were found to be significant, especially when considering the value of unpaid work. Additionally, this study estimated the lifetime costs of heart failure using both prevalence-based and incidence-based methods, providing valuable information for cost-benefit analyses and calculating return on investment.

The present study employed econometric methods to investigate the relationships between clinical and socio-demographic variables and their impact on heart failure costs. The results of this analysis provided significant insights into the factors influencing the costs of heart failure. Given the substantial economic burden of this disease on patients, it is crucial to develop support and insurance mechanisms that cover treatment costs and support lost productivity. While the government in Iran has made efforts to provide comprehensive coverage for individuals, there are still some individuals who lack insurance. This lack of insurance directly impacts the costs associated with heart failure, as it creates barriers to accessing timely and appropriate medical services, leading to worsened conditions and increased direct and indirect costs in the future. The Probit regression results from this study also highlight the statistically significant effect of insurance on cost occurrence. However, it is important to note that insurance only covers a portion of medical expenses, leaving significant indirect costs uncovered, which greatly impact individuals’ well-being and living.

## Limitations

Despite being a comprehensive cost-of-illness study on heart failure, there are some limitations that should be considered when interpreting the findings. Firstly, the sample for this study was limited to the northwest region of Iran (East Azarbaijan province) and may not be representative of the entire country. Although treatment tariffs and protocols are generally consistent across Iran, generalizing the results should be done with caution. Secondly, the study sample only included patients from public hospitals, and it would have been beneficial to include patients from private hospitals as well. Although the tariffs applied for services were proportional to the prices in the private sector and were analyzed accordingly, including private sector patients would have provided a more comprehensive perspective. Thirdly, the follow-up period in this study ranged from 2 to 6 months, which may not fully capture the one-year timeframe that was generalized. Ideally, patients would have been followed up for a full year, but time and resource constraints limited this possibility. Lastly, the incidence-based method used simulation and modeling techniques, which may have limitations compared to using real data and patient follow-up.

## Data Availability

The original contributions presented in the study are included in the article/**Supplementary Material**, further inquiries can be directed to the corresponding author.
