# Supplementary material for: Heart failure: a prevalence-based and model-based cost analysis
Source: Front Cardiovasc Med. 2023 Dec 1;10:1239719. doi: 10.3389/fcvm.2023.1239719 (PMC10722181; doi:10.3389/fcvm.2023.1239719)
Supplement: Supplementary file 1 [file Table1.docx]

| Percent | Frequency | Status | Variable |
| --- | --- | --- | --- |
| 21.1 | 106 | Class I | HF |
| 37.7 | 189 | Class II |  |
| 17.5 | 88 | Class III |  |
| 23.7 | 119 | Class IV |  |
| 61.1 | 307 | Male | Gender |
| 38.9 | 195 | Female |  |
| 61 | 306 | Yes | Comorbidity |
| 39 | 196 | No |  |
| 91.2 | 458 | Yes | Basic insurance |
| 8.8 | 44 | No |  |
| 42.4 | 213 | Yes | Complementary insurance |
| 57.6 | 289 | No |  |
| 3 | 15 | Single | Marital status |
| 76.1 | 382 | Married |  |
| 0.9 | 5 | Divorced |  |
| 20 | 100 | Spouse died |  |
| 39.2 | 197 | Illiterate | Level of education |
| 52 | 261 | Under & Diploma |  |
| 6 | 30 | Associate's degree - Bachelor's degree |  |
| 2.8 | 14 | Master's degree - PhD |  |
| 0 | 0 | Seminary education |  |
| 52 | 261 | Local | State of residence |
| 48 | 241 | Non-local |  |
| 72 | 361 | Yes | Household Head |
| 28 | 141 | No |  |

Table 2. Average and standard deviation of the annual costs of heart failure

| Variable | | | M  Mean (Toman) M | Standard deviation (SD) |
| --- | --- | --- | --- | --- |
| Average total annual cost | | | 261,409,854.9 | 369,357,786.7 |
| Average annual direct cost | **Medical** | **Medicine** | 5,053,339.627 | 31,551,902.47 |
|  |  | **Treatment** | 44,979,659.96 | 100,769,273.6 |
|  |  | **Diagnostic** | 2,184,442.024 | 6,206,215.425 |
|  | **Non-medical** | | 2,001,997.923 | 5,283,430.999 |
| Average annual indirect cost | **Premature death** | | 29,403,824.7 | 158,145,195.4 |
|  | **Absenteeism** | | 9,390,793.338 | 28,323,561.66 |
|  | **Presenteeism** | | 316,600.2596 | 2,292,269.639 |
|  | **Job change** | | 0 | 0 |
|  | **Unpaid work** | | 168,036,587.5 | 315,918,806 |
| Average annual cost | **Class I** | | 284,221,375.3 | 484,971,996.5 |
|  | **Class II** | | 184,586,207.1 | 309,046,776.5 |
|  | **Class III** | | 302،719,797.7 | 345,431,304.9 |
|  | **Class IV** | | 332,555,849 | 331,530,122.6 |

| Table 3. Two-part regression results (A. Discrete regression Probit, B.Continuous regression GLM) | | | | | | |
| --- | --- | --- | --- | --- | --- | --- |
| A.Discrete Total Cost  (Probit) | Coef | Std.Err | Z | P> IzI | Conf. Interval | |
| Age | -.0108625 | .0062124 | -1.75 | 0.080 | -.0230387 | .0013136 |
| Gender | -.1660696 | .1780375 | -0.93 | 0.351 | -.5150166 | .182877 |
| Base insurance | .6149499 | .2497434 | 2.46 | 0.014 | .1254619 | 1.104438 |
| Comorbidity | -.0449416 | .1918412 | -0.23 | 0.815 | -.4209435 | .3310603 |
| Disease Class | -.0960613 | .0830214 | -1.16 | 0.247 | -.2587803 | .0666576 |
| Cons | 1.988594 | .4829774 | 4.12 | 0.000 | 1.041975 | 2.935212 |
|  | | | | | | |
| B.Continuous regression (GLM) | Coef | Std.Err | Z | P> IzI | Conf. Interval | |
| Age | -.0011812 | .0046353 | -0.25 | 0.799 | -.0102663 | .0079038 |
| Gender | -.363384 | .1385386 | -2.62 | 0.009 | -.6349148 | .0918533 |
| Base insurance | -.3233732 | .241476 | -1.34 | 0.181 | -.7966576 | .1499112 |
| Comorbidity | .0577854 | .1357614 | 0.43 | 0.670 | -.2083021 | .3238729 |
| Disease Class | .1208097 | .0578797 | 2.09 | 0.037 | .0073676 | .2342518 |
| Cons | 19.60896 | .3910967 | 50.14 | 0.000 | 18.84242 | 20.37549 |
| Log linkelihood = -9538.861943, AIC = 40.87735, BIC= - 1835.199 | | | | | | |
